# Histone dynamics during DNA replication stress

**DOI:** 10.1186/s12929-021-00743-5

**Published:** 2021-06-19

**Authors:** Chia-Ling Hsu, Shin Yen Chong, Chia-Yeh Lin, Cheng-Fu Kao

**Affiliations:** Institute of Cellular and Organismic Biology, Academia Sinica, Taipei, 11529 Taiwan

**Keywords:** Replication stress, Genome instability, Histone dynamics, Histone modifications, Histone variants

## Abstract

Accurate and complete replication of the genome is essential not only for genome stability but also for cell viability. However, cells face constant threats to the replication process, such as spontaneous DNA modifications and DNA lesions from endogenous and external sources. Any obstacle that slows down replication forks or perturbs replication dynamics is generally considered to be a form of replication stress, and the past decade has seen numerous advances in our understanding of how cells respond to and resolve such challenges. Furthermore, recent studies have also uncovered links between defects in replication stress responses and genome instability or various diseases, such as cancer. Because replication stress takes place in the context of chromatin, histone dynamics play key roles in modulating fork progression and replication stress responses. Here, we summarize the current understanding of histone dynamics in replication stress, highlighting recent advances in the characterization of fork-protective mechanisms.

## Introduction

As replication forks proceed through the chromatin of eukaryotic cells, a large number of obstacles will be encountered, and these obstacles must be repaired or bypassed to ensure accurate duplication of DNA and maintenance of genome integrity. Barriers to replication may include secondary structures formed by certain DNA sequences, specific genome regions that are difficult to replicate, DNA lesions, chemically modified nucleotide bases, proteins tightly bound to DNA, DNA/RNA hybrids, or deficiencies in deoxyribonucleotide triphosphates (dNTPs) [1, 2]. These impediments to replication fork progression are potential sources of replication stress, and there is growing evidence that cells have evolved specific fork repair mechanisms to overcome each type of obstacle [3]. Some barriers cause replication forks to pause, followed by restart without fork collapse [4], while others cause stable stalling of replication forks until a converging fork arrives to mediate replication termination [5, 6]. However, the specific factors that determine the fate of a replication fork in response to a given obstacle remain unclear.

**Fig. 1:**
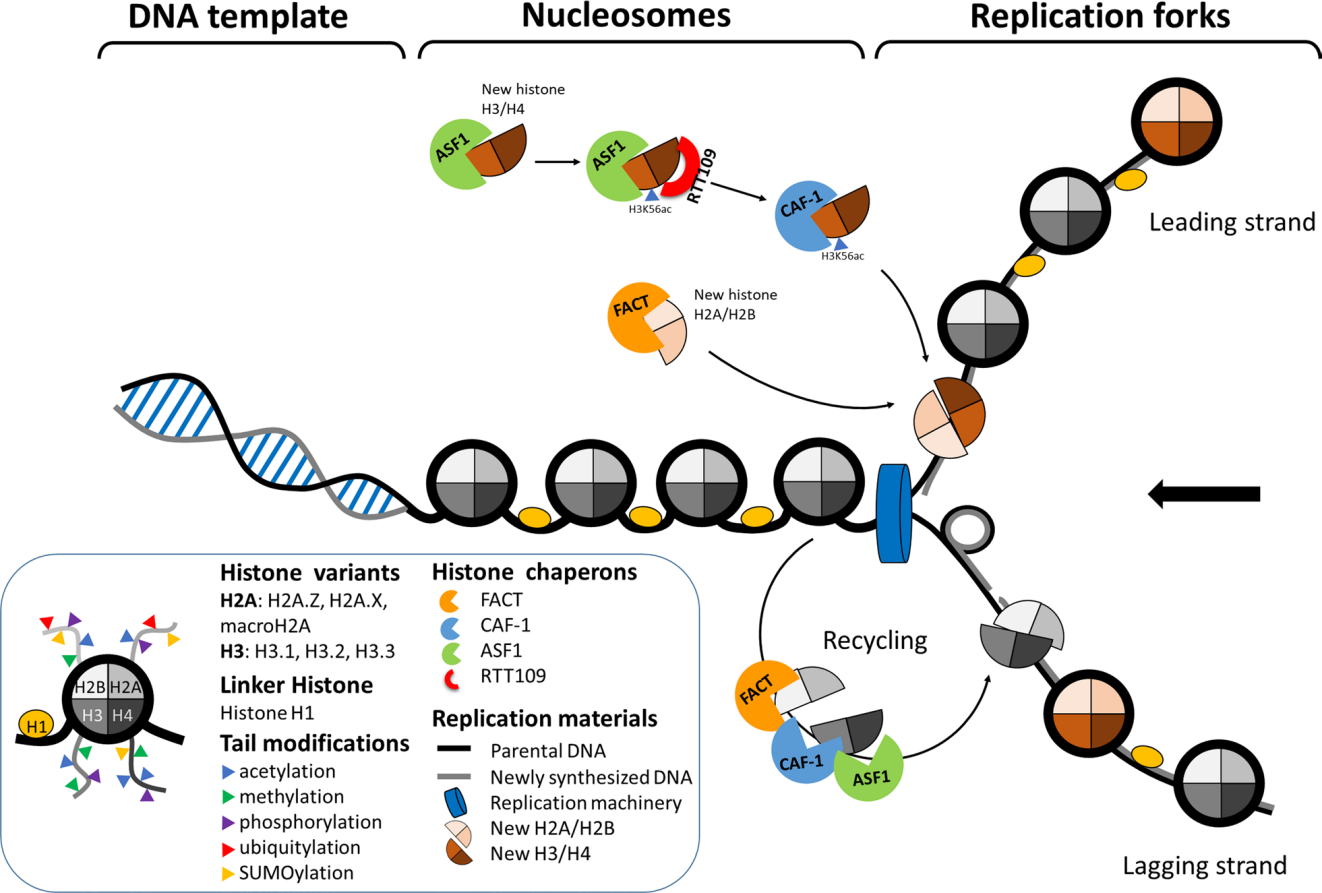
A simplified model for eukaryotic DNA replication forks. Unlike the situation in prokaryotes, eukaryotic DNA replication is carried out in the context of chromatin. Replication is initiated at multiple distinct replication origins along the chromosome. Unwinding of the double-stranded DNA at origins allows for assembly of a specialized structure called the replication fork (resembling a two-tined fork), where a large group of replication proteins (replication machinery) are dynamically coordinated to duplicate the genome. Importantly, there is a crucial interplay between the replication machinery and chromatin dynamics (including histone eviction and recycling at the fork, specific post-transcriptional modifications, and exchange of canonical histones with histone variants via histone chaperones). When replication forks encounter obstacles that block replicative DNA polymerases and induce fork stalling (replication stress), chromatin structural components may contribute to repair/checkpoint machineries that rescue cells from replication stress

Importantly, eukaryotic DNA replication is carried out in the context of chromatin. The fundamental unit of chromatin is the nucleosome, which consists of a segment of DNA wrapped around a core of histone proteins. Physical interactions between the nucleosome and the replisome (a multi-protein molecular machinery responsible for DNA replication) are known to occur. The current view is that an active replisome will evict parental histones ahead of the machinery, and the evicted histones will be recycled into newly replicated DNA, along with newly synthesized histones [7]. This process is mediated by various histone chaperones, such as anti-silencing factor 1 (ASF1), chromatin assembly factor 1 (CAF-1), facilitates chromatin transcription (FACT) and RTT109 [8] (Fig. 1). Following the chaperone-mediated assembly of nucleosomes, their compaction levels, positions, and even variant histone compositions are then further altered by chromatin remodelers. Moreover, as key mediators of efficient cellular responses to replication stress, histone chaperones and chromatin remodelers are necessary for genome maintenance and stress tolerance. The molecular details of these processes have been extensively reviewed elsewhere [9–11], and thus, we do not repeat the information here, except as it pertains to histone modifications or histone variants.

Interestingly, specific post-transcriptional modifications (PTMs) on parental and newly synthesized histones flanking replication forks have been shown to coordinate with core components of various repair mechanisms or different checkpoint machineries. The histone PTMs are regulated by protein machineries that ‘write’, ‘read’ and ‘erase’ the histone marks, called histone writers, readers and erasers [12]. The PTM-containing histones generally serve to facilitate access of specialized repair or checkpoint proteins to replicating chromatin when replication barriers are encountered (Fig. 1). Furthermore, differential histone variant exchange has also been associated with replication stress response. Such exchanges can generate a microenvironment to facilitate recruitment of accessory fork factors throughout large chromatin domains (Fig. 1). In this review, we introduce the latest advances in characterizing the repair/checkpoint machineries that rescue cells from replication stress, emphasizing the important roles of histone variants and PTMs in replication stress response.

## Replication stress

### Formation of the eukaryotic replication fork

In eukaryotes, DNA replication is initiated at multiple individual replication origins, and its licensing involves recruitment of the origin recognition complex, multiple other proteins, and the loading of MCM2-7 helicase [13, 14]. Together, these factors constitute pre-replicative complexes (pre-RCs). In budding yeast, replication origins are associated with AT-rich elements called autonomously replicating sequence (ARS) consensus sequences (ACSs) [13]. On the other hand, metazoans lack a specific origin sequence, and the origin sites are thought to be determined by a combination of DNA sequence and chromatin-associated factors [15]. Moreover, recent evidence suggests that origin recognition may be regulated by epigenetic signatures. Histone variant H2A.Z is broadly enriched at replication origins [16, 17] and was shown to play a functional role in recruiting the histone lysine methyltransferase enzyme, SUV420H1; this action promotes H4K20me2 deposition at origins and regulates the licensing and activation of early replication origins through interactions between H4K20me2 and ORC1 [18]. Interestingly, eukaryotic origin positioning may be more dynamic than previously thought, as origins can be shifted by the sliding of MCM2-7 complexes along the chromosomes due to collisions with RNA polymerase [19].

In the pre-RC, the MCM2-7 helicase is inactive and unable to unwind double-stranded DNA. At the G1/S-phase transition, DBF4-dependent kinase (DDK) and CDK phosphorylate specific sites in pre-RCs, facilitating the recruitment of CDC45 and GINS complex to form the CMG complex (CDC45-MCM-GINS), which has active replicative helicase. Additional factors cooperate with the active helicase to unwind the DNA and further recruit other replication factors, such as replication factor C (RFC), replication protein A (RPA), the sliding clamp proliferating cell nuclear antigen (PCNA), and multiple DNA polymerases, known collectively as the replisome [20]. With assembly of the replisome, two replication forks are established and may progress in opposite directions from the activated origin [21, 22]. It is worth noting that only a subset of all licensed origins are activated during a given S phase, with the rest remaining dormant (licensed but not activated), ready to provide backup in certain conditions, such as replication stress [1].

### Sources of replication stress

Perturbations in replication fork progression and/or DNA synthesis lead to the accumulation of extended single-stranded DNA (ssDNA) tracts at replication forks, which represents the primary signal to trigger replication stress response. The ssDNA tracks are generated as the helicase continues to unwind adjacent DNA, while progression of the replication fork is slowed down or stalled [23]. There are several known endogenous and exogenous causes of helicase-polymerase uncoupling. First, obstacles on the DNA template can directly impede advancing replication forks. These impediments may include DNA lesions induced by UV light or chemical mutagens [24], oxidized or abasic sites resulting from excess reactive oxygen species (ROS) [25], DNA secondary structures formed at repetitive nucleotide repeats, or unique DNA structures, such as stem-loops and G-quadruplexes formed at AT- and GC-rich regions [26]. In addition, incorporation of ribonucleotides into DNA by Polϵ (epsilon) variants can also create barriers to replication fork progression [27].

Certain levels of dNTPs are necessary for DNA replication [28], and dNTP deficiencies are a source of replication stress. In fact, it has been shown that dysregulation of proliferation-regulating oncoproteins contributes to replication folk stalling, DSBs and oncogene-induced transformation via insufficient dNTP supplies [29]. Moreover, oncogene activation can induce replication stress by directly interfering with nucleotide biosynthesis [30].

Active replication forks often encounter transcriptional machinery, as the two processes utilize the same DNA template. Collisions of replication and transcription complexes may occur in two orientations: co-directional (CD) conflicts involve replication forks progressing in the same direction as the transcription machinery, while head-on (HO) conflicts involve collisions of the two machineries operating in opposite directions. As HO collisions are generally thought to be more disruptive than CD collisions, higher organisms appear to have evolved in such way that replication is frequently initiated near the start sites of highly transcribed genes, which ensures that the replication forks move through transcribed regions of the genome in an orientation that creates bias toward CD collisions [31]. Interestingly, human cells exhibit a global reorientation of replication relative to transcription around the 3’ ends of genes upon replication stress, leading to increased incidence of HO collisions, i.e., major transcription-replication conflicts (TRCs) [31]. Additionally, chromatin conformation may participate in coordinating the dynamics of DNA replication and transcription, as a more open chromatin structure due to low histone-DNA ratios was shown to induce TRC-mediated replication stress and DNA damage signaling [32].

The R-loop is an especially noteworthy structure on the DNA template. Although R-loops are prevalent and dynamically formed under physiological conditions [33], these structures are highly associated with TRCs, especially HO TRCs [34]. R-loops are generated by re-annealing of a nascent transcript to the transiently accessible DNA duplex behind RNA polymerase, resulting in an RNA:DNA hybrid, with the non-transcribed DNA strand left to loop out. If such a structure persists, it can act as a potent obstacle to replication fork progression, leading to replication stress or sensitizing the genome to DNA damage due to accumulation of ssDNA tracts [35, 36]. Chromatin structure and modifications have been reported to ensure smooth DNA replication by preventing the formation of R-loops [37, 38]. However, it should be emphasized that although the formation of R-loops is associated with detrimental HO TRCs, R-loops are necessary for many normal physiological processes, such as DNA methylation [39], histone modifications [40], regulation of transcription termination [41], and chromosome segregation [42]. Thus, cells have evolved various strategies to tightly regulate R-loop dynamics [43–45].

In this review, we have limited our discussion of replication stress to the slowing or stalling of replication fork progression during DNA synthesis as result of various insults. Thus, other important types of replication defects, such as re-replication, over-activation of origins or under-usage of origins, are not discussed. Nevertheless, these scenarios may sensitize cells to many of the replication stress sources mentioned above [46, 47]. Similarly, activation of oncogenes commonly contributes to replication stress. Generally, the mechanisms of oncogene-induced replication stress revolve around the mechanisms mentioned above, and DNA replication stress is now considered to be a hallmark of cancer [48]

### Resolution of replication stress

**Fig. 2:**
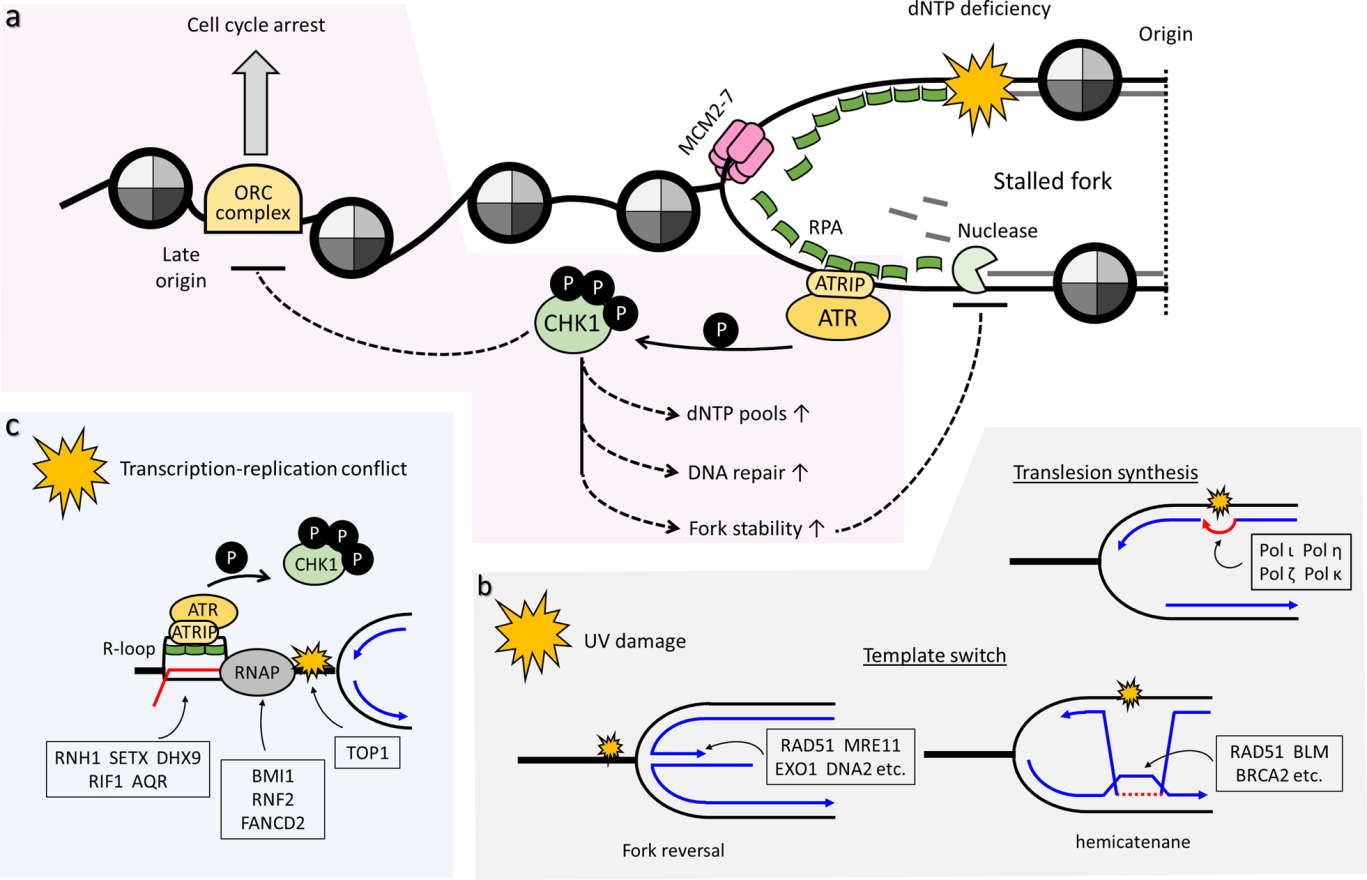
Resolution of replication stress. Impediments to replication fork progression lead to the generation of extended ssDNA tracts that initiate replication stress response. Recognition of ssDNA tracts by RPA serves as a signaling platform to trigger the ATR-CHK1 pathway (**a**). Once activated, this pathway triggers cell cycle arrest by inhibiting late origin firing. At the same time, activated CHK2 kinase acts through downstream effectors to promote processes crucial to restarting the stalled replication and preventing fork collapse, such as elevated dNTP production, DNA repair and nuclease activities. Lower illustrations show schematic representations of how replication stress may be resolved by indicated key factors; resolution of UV damage (**b**) and TRCs (**c**) are depicted

Diverse cellular mechanisms have evolved to maintain genome stability during DNA replication by responding to and resolving replication stress (Fig. 2). Despite the fact that replication stress may be triggered by various mechanisms, the triggers usually impinge on generation and accumulation of ssDNA molecules via impaired function of MCM2-7 helicase and DNA polymerase, either as a consequence of delays in polymerase progression or from DNA end-resection due to replisome pausing [1, 23]. The tracts of ssDNA are recognized by replication protein A (RPA), and if the resulting complex persists, it serves as a signaling platform to recruit the ataxia telangiectasia-related kinase (ATR)-interacting protein (ATRIP) (Fig. 2a). After ATRIP binds the RPA-ssDNA complex, ATR kinase is recruited to coordinate multiple checkpoint pathways at the site of replication stress [49]. As part of its function, ATR directly phosphorylates checkpoint kinase 1 (CHK1), along with other proteins including histone variant H2A.X (γH2A.X) and RPA [49]. Once activated, the ATR-CHK1 pathway works to alleviate replication stress and preserve genome stability by inhibiting late origin firing and cell cycle progression (Fig. 2a). The main purpose of these events is ostensibly to provide extra time for resolution of the stress and to enable the concentration of DNA synthesis resources at sites near the stress [1]. Simultaneously, ATR also promotes the stabilization and restart of stalled replication forks via a variety of mechanisms. These mechanisms include initiation of replication from dormant origins, fork reversal (or fork regression), and the activation of DNA damage tolerance pathways involving template switching or specialized translesion synthesis (TLS) polymerases, such as Polη (eta), Polκ (kappa), Polι (iota), Polζ (zeta) and Rev1 [1, 50] (Fig. 2b).

Among the ATR-stimulated effects on replication forks, fork reversal is a key response in which a typical three-way replication junction is remodeled into a four-way Holliday junction (HJ)-like structure [51]. This process is recognized as a global response to replication stress in metazoans, wherein fork reversal acts as an ‘emergency brake’ to transiently protect the cell from further damage (Fig. 2b). The transient HJ-like structure limits continued synthesis on lesion-containing templates, which might otherwise accumulate DSBs, and it prevents excessive accumulation of ssDNA, allowing more time and space for the repair machineries to operate [4]. Moreover, the HJ-like structure may promote template switching for error-free DNA synthesis [51], and an HJ-like structure with a DNA-end resembling a DSB can recruit HJ resolvases (e.g., BLM), homologous recombination (HR) factors (e.g., RAD51), and DSB repair factors (e.g., BRCA2) [51, 52]. Despite the fact that fork reversal is important for maintenance of genome stability, the regressed arms of reversed forks are highly susceptible to several nucleases, including MRE11, EXO1, DNA2, and CtIP [51]. Uncontrolled fork degradation by these nucleases may lead to fork collapse, increased genome instability, and even chemotherapy resistance of tumor cells [53]. Therefore, mechanisms to prevent excess nuclease-mediated degradation of nascent DNA are also required for replication fork stabilization. The components of the Fanconi anemia (FA) and HR pathways include RAD51 (FANCR), FANCD2, BRCA1 (FANCS) and (FANCD1), and these pathways were shown to coordinately suppress nascent DNA degradation [54]. However, the underlying mechanisms whereby these enzymes protect nascent DNA from degradation are still not well understood. Additionally, homologous recombination repair and break-in replication repair pathways may be activated at stalled replication forks, under conditions where fork collapse occurs and generates single-ended DSBs [55, 56].

As mentioned, TRCs and unscheduled formation of R-loops impede fork movement and represent an active area of research on replication stress and genome instability. Increasing numbers of studies have pointed out the many strategies used in cells to prevent TRCs or to remove R-loops (Fig. 2c). For example, R-loops, once formed, can be digested by RNase H 5’-3’ exonucleases, such as RNaseH1 (RNH1), or the structures can be resolved by specific helicases, such as DHX9, Aquarius (AQR), senataxin (SETX) and RIF1 [57, 58]. Moreover, R-loop formation at terminators of highly expressed genes can be prevented by topoisomerase I-mediated relaxation of DNA supercoiling [59]. In addition, TRCs and DNA:RNA hybrids activate the Fanconi anemia (FA) DSB pathway to resolve R-loops [60, 61]. In line with this mechanism, a recent study showed that SLX4, a tumor suppressor, directs the recruitment of FANCD2 (a critical FA complex member) to RNA polymerase II, and this action is necessary for prevention of TRCs in unstressed cells [62]. Polycomb group proteins BMI1 and RNF2 were recently revealed to suppress TRCs as well [63]. Lastly, transcription-coupled R-loops can also be resolved by RNA exosomes [64], RPA function [65], and the ATR-CHK1 pathway [44].

## Histone dynamics in replication stress

### Nucleosomes: the building blocks of chromatin structure

Eukaryotic genomes are packaged into nucleosomes, the basic units of chromatin, in order to balance fitting the DNA inside the tight confines of the nucleus while still retaining accessibility for transcription and replication. This balance can be achieved because nucleosomes support highly dynamic chromatin structure via their composition, conformation, and modulation by specialized enzymes. The nucleosome consists of 147 bp of duplex DNA wrapped around a core octamer of histone proteins. Each octamer contains two molecules each of four different histone proteins: H3 H4, H2A and H2B. These core histones all contain a conserved C-terminal hydrophobic histone fold domain (HFD) that is essential for inclusion in the nucleosome. The HFD mediates the formation of H2A-H2B and H3-H4 heterodimers that can then undergo tripartite modular protein assembly; two (H3-H4) heterodimers interact to form a tetramer that binds the inner turn of DNA (~ 70–80 bp), while two (H2A-H2B) heterodimers dock on both sides of the tetramer with the remaining ~ 40 bp of DNA wrapped on each end [66, 67]. The nucleosome core is compact, and its detailed atomic structure has been solved by X-ray crystallography and cryogenic electron microscopy [67, 68]; however, the positively charged N(C)-terminal tails that extend from the core are flexible and accessible to modifying enzymes, so the structures remain elusive. Recently, the dynamics and post-translational modifications (PTMs) of histone tails have been studied in detail using NMR spectroscopy [69, 70]. These studies have established that histone tails adopt distinct dynamic states that are able to regulate one another, probably creating a histone tail network inside the nucleosome.

### Roles of histone modifications in replication stress response

**Fig. 3:**
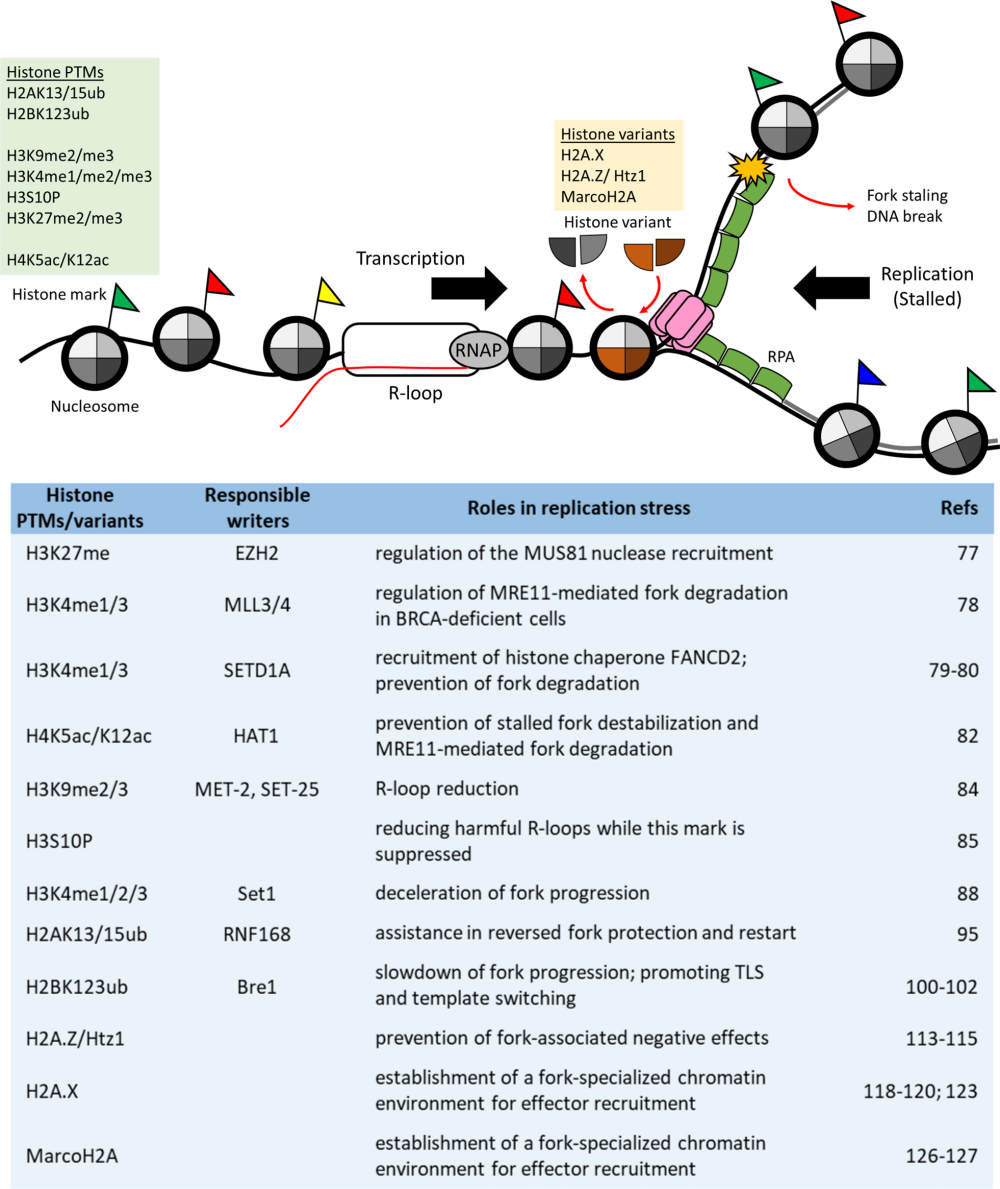
Histone dynamics during replication stress. Selected functions of histone modifications and variants in resolving replication stress are shown. Histone marks and the corresponding catalytic protein(s) are listed along with their main function in resolving certain types of replication stress. Certain histone variants are incorporated in response to stalled fork signals to facilitate fork restart. These events are certain to be highly coordinated with one another

While the complex role of chromatin in DNA replication has been appreciated for many years [71], it is now also becoming apparent that crucial aspects of replication stress response are linked to chromatin as well. New proteomic tools, including iPOND (isolation of proteins on nascent DNA) and NCC (nascent chromatin capture), have been invented in the past few years to facilitate the purification, identification, and quantification of chromatin maturation and replication stress response machineries [72]. These advances have led to discoveries of novel chromatin-related proteins and factors involved in replication stress and provided insights into histone dynamics around replication forks. In this section, we summarize the most recent advances in our understanding of how the chromatin environment, particularly with regard to histone modifications and variant histones, influences key aspects of replication stress response (Fig. 3).

#### Modifications of H3 and H4 involved in stalled fork degradation

To ensure the completion of DNA replication and maintenance of genome integrity, cells have evolved an elaborate network of replication stress responses that protect stalled replication forks. An increasing number of studies show that chromatin-related proteins are involved in this process. For example, SNF2-family ATP-dependent chromatin remodeling factors, including SMARCAL1, ZRANB3 and HLTF, are required for MRE11-dependent degradation of stalled DNA replication forks in BRCA1/2-deficient cells [73]. Moreover, serveral chromatin modifiers and their associated histone modifications are thought to participate in the prevention of fork degradation.

##### EZH2 and H3K27me3

 The PRC2 subunit, enhancer of zeste homolog 2 (EZH2), is a lysine methyltransferase (KMT) that mediates H3 lysine 27 di- and tri-methylation (H3K27me2/me3) in normal DNA replication [74, 75]. Recently, the use of a modified version of iPOND, accelerated native isolation of protein on nascent DNA (aniPOND) [76], on cells with fork stalling after hydroxyurea (HU) treatment revealed that levels of EZH2 and H3K27me3 were increased; the increases were consistent with enhanced EZH2 activity at stalled forks, and the local spread of H3K27me3 was linked to recruitment of MUS81 nuclease [77]. This work identified EZH2/H3K27me3 as a important regulator for genome stability in BRCA2-deficient cells. In BRCA2-mutated tumors, low EZH2 levels prevent MUS81 recruitment, which in turn enhances fork protection. Accordingly, loss of function in the EZH2/H3K27me3/MUS81 axis may serve as a predictive biomarker for chemoresistance in cancer patients with BRCA2 deficiency.

##### MLL3/4 or SET1A (the mammalian homologues of yeast Set1) and H3K4me

 Apart from EZH2, other KMTs (i.e., MLL3/4 histone methyltransferases that catalyze H3 lysine 4 methylation [H3K4me] and establish H3K4me1/me3 at replication forks) were found to promote MRE11-mediated fork defgradation in BRCA-deficient cells [78]. Interestingly, recent work shows that H3K4me deposited at stressed forks by the KMT, SETD1A, directs recruitment of the FA protein, FANCD2, enhancing FANCD2-dependent histone chaperone activity [79]. Since this chaperone activity is required for the stabilization of RAD51-mediated nucleofilaments and prevention of fork degradation, loss of function in the SETD1A/H3K4me1/FANCD2 axis sensitizes cells to replication stress and leads to DNA2-dependent fork resection [79]. This work also highlights how dynamic chromatin remodeling processes at stressed forks can prevent genome instability. Similarly, it was shown that during replication stress, yeast cells have a requirement for the KMT, Set1, a subunit of the evolutionarily conserved enzyme complex that catalyzes H3K4me1/me2/me3 deposition and is called complex proteins with Set1p (COMPASS) [80].

##### HAT1 and H4K5ac/K12ac

 Using iPOND, histone acetyltransferase 1 (HAT1), which is responsible for the cytosolic diacetylation of newly synthesized H4 on lysine 5 and 12, was shown to transiently associate with newly replicated DNA [81]. Interestingly, this transient association can be stabilized by replication fork stalling and may be functionally linked to proper replication fork function and stability [82]. Loss of this modification on newly synthesized H4 HAT1 causes alterations in nascent chromatin structure at stalled forks, which lead to destabilization of stalled forks and MRE11-dependent degradation of newly synthesized DNA [82]. This work not only expands our understanding of the role of HAT1 to include genome stability, but it also suggests an update should be made to current models of replication-coupled chromatin assembly to incorporate the localization of HAT1 to nascent chromatin near DNA replication sites.

Together, these recent studies suggest that histone PTMs can be promoted at stalled replication forks by different factors to stabilize the stressed replication fork. These results indicate that potential epigenetic mechanisms serve as a platform for the recruitment of appropriate replication stress response proteins. Understanding the regulation of histone-modifying pathways during distinct replication stress events will be critical to gain insights into their role in fine-tuning diverse cellular stress responses.

#### The roles of H3 modifications in the resolution of TRCs

##### H3K9me and H3S10P

 R-loops have major regulatory roles in gene expression. Hence, it is not surprising that active histone marks are correlated with R loop formation [83]. Transcription-elongation-coupled H3K9 methylation (H3K9me) suppresses R-loop-associated genome instability at repeated sequences in *C. elegans* by reducing transcription of heterochromatin repeats, sheilding the replication process from potential interference by TRCs [84]. Based on their data from a yeast genetic screen, Aguilera’s lab suggested that a two-step mechanism may explain why R loop-mediated genome instability is correlated with chromatin modifications [85]. They proposed that R-loops *per se* do not cause genetic instability; however, R-loops may trigger local chromatin remodeling that can serve as a barrier to DNA replication. The phosphorylation of serine 10 in histone H3 (H3S10P) is known to play a role in R-loop-correlated hyper-recombination, and the accumulation of this histone modification is triggered by R-loops, possibly also causing the chromatin to become more condensed [85]. Although the H3S10P chromatin mark is known to be associated with chromatin condensation in mitosis and meiosis [86, 87], the molecular linkage is still unknown. Thus, resolving the mechanisms by which R loops stimulate H3S10P and understanding how this histone PTM promotes chromatin condensation will be critical for establishing a causal link between the two processes.

##### Set1 and H3K4me

 Transcription-induced H3K4me is able to decelerate active replication forks, and this function may help to prevent the occurrence of catastrophic TRCs, especially in highly transcribed genes [88]. However, the mechanistic details of H3K4me-mediated fork deceleration remain undefined. H3K4me3 is recognized by a PHD finger within the ING family of proteins (ING1-5) [89]. H3K4me3 is also bound by the tandem chromodomains within CHD1, an ATP-dependent remodeling enzyme capable of repositioning nucleosomes [90], and by the tandem Tudor domains within JMJD2A, a histone demethylase [91]. Thus, one possible mechanism for H3K4me to regulate fork progression is through recruitment of some reader complex to the nucleosome, which could either create a physical barrier or limit histone eviction efficiency.

Alternatively, H3K4me may decelerate ongoing replication by influencing genome topology.

Chromosome folding analysis in budding yeast, using a Hi-C-based method called Micro-C, uncovered abundant chromosome interaction domains (CIDs), which are similar to the reported topologically associating domains (TADs) in mammals [92]. Strong boundaries between CIDs occur at promoters of highly transcribed genes. Intriguingly, nucleosomes at the boundaries exhibit significant enrichments of a variety of histone marks at the 5’ ends of genes [93], including high levels of transcription-related marks such as H3K4me3 and H3K18ac. Furthermore, deficiency of Cfp1, a conserved subunit of the Set1 complex in mouse, causes a shift of H3K4me3 from the promoters of expressed genes to numerous “ectopic sites”; however, this disruption has minimal consequences on transcription. Further analysis revealed that these ectopic peaks are enriched for cohesin and CTCF binding sites, which are thought to mediate chromatin looping [94]. Together, these results imply a role for H3K4me in chromatin organization. Furthermore, global genetic analysis of gene pairs in yeast reveals that deletion of *SET1* positively interacts with mutations in subunits of cohesin and condensin [95], which suggests a functionally proximal relationship between the proteins. Thus, further investigations in this direction may reveal a possible functional role for H3K4me in regional and/or global chromatin organization that might influence DNA replication.

#### Modifications of H2A and H2B involved in stalled fork reversal and protection

##### H2Aub

 While H3 methylation has been implicated in the closely linked processes of replication fork reversal and protection, other studies suggest that ubiquitination of both H2A and H2B may also be required for the resolution of replication stress. Ubiquitination of H2A lysine 13 and lysine 15 (H2AK13/15ub), mediated by the E3 ubiquitin ligase RNF168, was recently found to be important for efficient fork progression [96]. It has long been known that this pair of modifications is essential for activation of downstream DNA damage signalling and DNA repair [97]. However, a recent study showed that loss of the RNF168/H2AK13/15ub axis also causes slow fork progression and reversed fork accumulation at difficult-to-replicate sequences. This delayed fork progression requires MRE11-dependent degradation of reversed forks, implicating RNF168/H2AK13/15ub in reversed fork protection and restart. These data thus imply that RNF168 and other factors in the DNA damage response (DDR) signalling pathway can be recruited to a reversed fork, probably due to its DSB-like DNA end, and this recruitment is required to prevent reversed fork accumulation and degradation by MRE11 [96]. More recently, a novel histone mark, the phosphorylation of ubiquitin threonine 12 on H2AK15ub, was identified as mediator of the DDR signalling cascade [98]. A comprehensive NCC assay to analyze the proteomic profile of replication forks challenged by topoisomerase 1 (TOP1) inhibition (including the chromatin environment) revealed a novel framework for repair of broken replication forks. Based on this analysis, the authors of the study were able to conclude that the RNF168/H2AK15ub response is suppressed at broken forks to promote ATM and PLK1 (Polo-like kinase-1)-mediated HR [99]. It will be thus be interesting to further determine whether ATM signaling might be involved the phosphorylation of H2AK15ub and to define the molecular actions of these sequential PTM events in preventing genome instability.

##### H2Bub

 In yeast, ubiquitination of H2B lysine 123 (H2BK123ub) has been linked to replication stress signaling in several reports [100–102]. These studies have all shown that the absence of H2BK123ub leads to replication stress and defective replication fork progression, but each report provides a different mechanistic explanation. In one model, H2Bub is thought to play a role similar to a bump in the road, serving to slow down fork pregression and presumably allow cells more time to repair DNA lesions at stressed forks [102]. On the other hand, a study on DNA damage tolerance upon fork stalling suggested that H2BK123ub may be required to promote TLS by DNA polymerase Polη (eta) and Polζ (zeta) [101]. In accordance with this idea, H2BK123ub aids template switching and HR to bypass DNA lesions [100]. Surprisingly, the function of H2BK123ub in lesion bypass is important not only during DNA replication but also after replication [100]. The role of H2BK123ub in post-replication DNA damage repair is intriguing, as the mechanistic basis is largely unexplored. One possibility is that H2BK123ub in chromatin may further promote G2/M checkpoint activation to maintain stability and facilitate the filling of unrepaired ssDNA gaps.

In summary, histone PTMs appear to be important factors in the mitigation of replication stress. Each PTM seems to exhibit functional relevance in particular cellular contexts or upon certain replication stress-inducing stimuli.

### Histone variants in replication stress response

Although the nucleosomal core of canonical histones exhibits a highly conserved overall structure, several histone variants have been shown to increase the diversity and dynamics of the nucleosome and play essential roles in epigenetic regulation [103, 104]. In humans, several variants of H2A and H3 exist, while H2B has only a few variants, and only one form of H4 has been identified [105, 106].

#### H2A.Z

 Of all the variant histone subunits, three H2A variants are of particular relevance to replication stress; these include H2A.Z, H2A.X, and macroH2A. H2A.Z is one of the most evolutionarily conserved H2A variants, and it is typically enriched at the boundaries of nucleosome-depleted regions surrounding active promoters, where it promotes transcriptional activation [107]. Apart from its role in transcription regulation, multiple studies have implicated Htz1 (yeast H2A.Z) and its regulatory complex, SWR-C (Swi2/Snf2-related chromatin remodeling complex), in maintaining genome stability [108–112]. Recently, the potential importance of SWR-C/Htz1 in replication stress was revealed, as Htz1 deposition and retention in chromatin by SWR-C were found to prevent transiently stalled replication forks in replication-fork-checkpoint-defective mutants from being converted to DNA DSBs [113]. Furthermore, Ino80-mediated removal of Htz1 also affects genome stability through both DDR and replication stress pathways [114, 115]. Thus, it seems that H2A.Z dynamics are orchestrated to prevent negative effects of stalled replication forks, which is clearly important for genome maintenance.

#### H2A.X

 In addition, H2A.X is known to be loaded near DSB sites, and it is phosphorylated by DNA damage checkpoint kinases at Ser139 inside its characteristic C-terminal SQE motif to produce phospho-H2A.X (γH2A.X) [116]. Of note, in yeast, the phosphorylation of canonical H2A at Ser129 is functionally similar to γH2A.X, whereas in *Drosophila*, a single bi-functional variant, H2A.v, has the properties of both H2A.Z and H2A.X [117]. Since γH2A.X can be generated by three kinases that respond to various types of DNA damage throughout the cell cycle, it is not considered to be a specific marker of replication stress. Nevertheless, upon replication stress, the phosphorylation of H2A.X is carried out by ATR [118], one of the central replication-stress response kinases; in ATR-deficient cells, this phosphorylation is mediated by the other two kinases, ATM and DNA-PKcs [119]. Once phosphorylated, γH2A.X marks stalled replication forks prior to DSB formation [120], presumably to establish a chromatin environment that favors the recruitment of repair proteins. The accumulation of γH2A.X also occurs at DSB sites if a fork collapses, where it functions to promote DSB repair [120–122]. In addition, the importance of γH2A.X in rescuing stalled replication has been suggested by experiments in the yeast model [123]. Recently, ChIP-seq to map the γH2A.X distribution genome-wide caused by distinct fork stalling mechanisms in a human lymphocyte cell line revealed that different treatments can induce non-random γH2A.X chromatin binding at discrete regions [124]. Characterization of the γH2A.X distribution showed two consistent epigenetic features: (1) different treatments induce γH2A.X loading at largely non-overlapping regions, and (2) γH2A.X loading hotspots are depleted at CpG islands and transcription start sites but are enriched at compact chromatin regions. The γH2A.X histone variant may therefore coordinate with different protein molecules and repair pathways to rescue forks stalled at different types of fragile sequences [124].

#### MacroH2A

MacroH2A, an H2A variant with an unusual C-terminal non-histone domain (i.e., macro domain), has also been shown to promote genome stability as an epigenetic mediator of replication stress response [125]. Conditions of replication stress induce the accumulation of macroH2A at fragile sites, which may serve as a platform for recruitment of repair proteins, reminiscent of the role for γH2A.X in DDR. In this case, BRCA1 is thought to be a key downstream effector due to a specific interaction between its N-terminal region and macroH2A [125]. Interestingly, the same group further showed that the macroH2A deposition requires the histone chaperone FACT (facilitates chromatin transcription), which also functions in the resolution of R-loop-mediated TRCs [126]. Furthermore, macroH2A is highly enriched at telomeres undergoing ALT (alternative lengthening of telomeres; a homology-directed telomere-maintenance pathway) in tumor cells [127]. Consistent with the inherent susceptibility to replication stress in ALT-deficient cells, during acute stress, the DDR-dependent dynamics of macroH2A at telomeres promote the execution of ALT; this work suggests macroH2A may be a potential therapeutic target for preventing tumor growth via manipulation of ALT [127].

Taken altogether, these studies show that during replication stress, histone variants play various roles in shaping specific chromatin structures according to the type of stress encountered, thus facilitating a more specifically targeted replication stress response.

## Conclusions

Studies over the past decade have provided important mechanistic insights into how cells resolve replication stress. It is now understood that wide variety of cellular surveillance events are coordinated to ensure faithful duplication of genome. The importance of these processes is highlighted by the fact that cancer cells display persisent replication stress, due to failures in protecting and repairing stalled replication forks during uncontrolled cell proliferation. This key difference between cancerous and healthy cells makes replication stress a promising target for anti-cancer therapies. Since replication stress occurs in the context of chromatin, advances in the understanding of how histone dynamics are coupled to replication stress might expand the array of replication stress response factors that can be targeted by novel therapeutics. The further discovery of potential drug targets may also reveal novel regulatory pathways involved in fork stabilization. Importantly, cancers carrying mutations that induce replication fork instability or compromise replication stress response pathways may be susceptible to treatments designed to exploit epigenetics-based synthetic lethality.

## Data Availability

Not applicable.
